# Supplementary material for: Functional signatures of ex-vivo dental caries onset
Source: J Oral Microbiol. 2022 Sep 19;14(1):2123624. doi: 10.1080/20002297.2022.2123624 (PMC9518263; doi:10.1080/20002297.2022.2123624)

## Slide 1
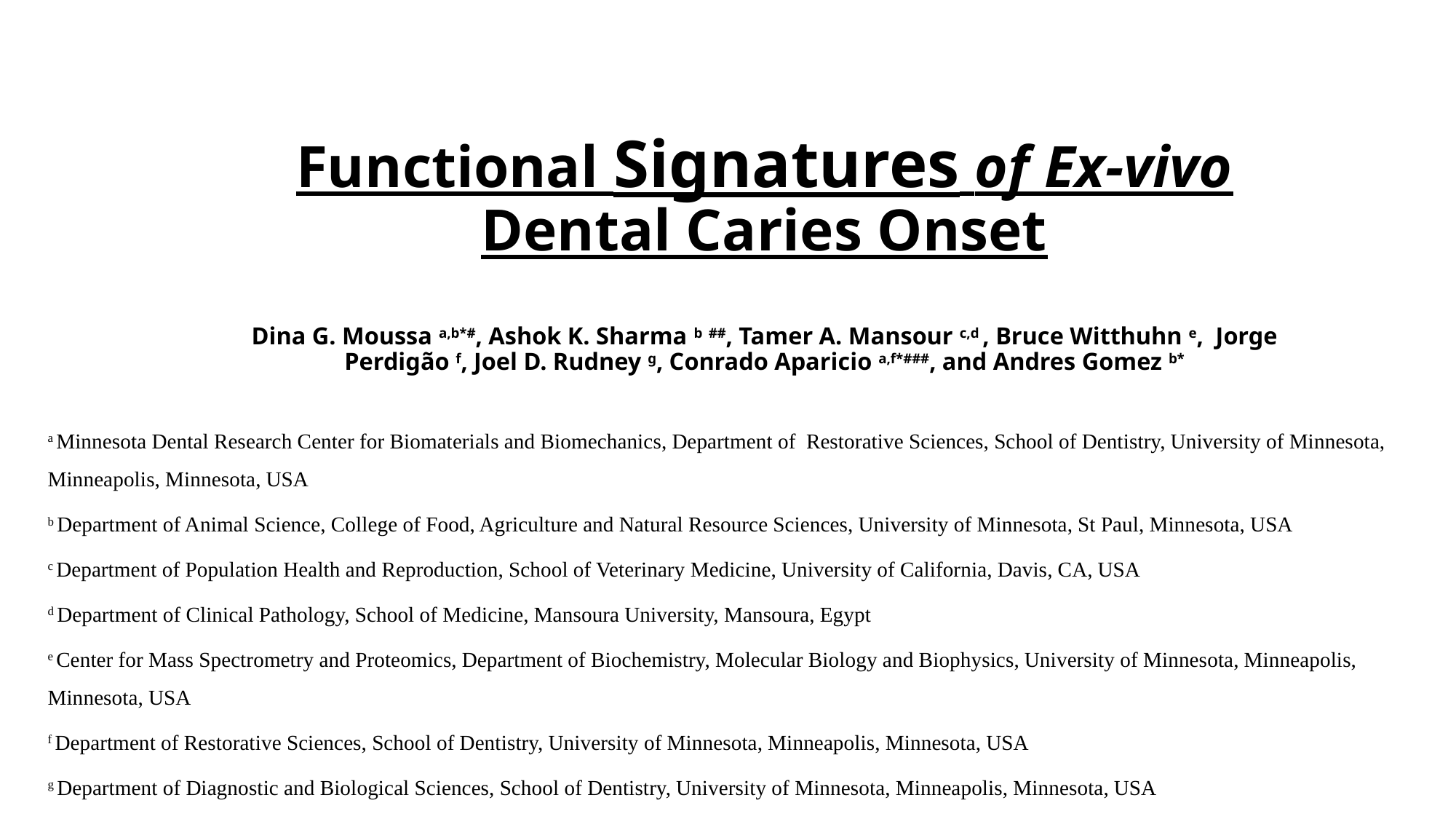

# Functional Signatures of Ex-vivo Dental Caries Onset Dina G. Moussa a,b*#, Ashok K. Sharma b ##, Tamer A. Mansour c,d , Bruce Witthuhn e, Jorge Perdigão f, Joel D. Rudney g, Conrado Aparicio a,f*###, and Andres Gomez b*
a Minnesota Dental Research Center for Biomaterials and Biomechanics, Department of Restorative Sciences, School of Dentistry, University of Minnesota, Minneapolis, Minnesota, USA
b Department of Animal Science, College of Food, Agriculture and Natural Resource Sciences, University of Minnesota, St Paul, Minnesota, USA
c Department of Population Health and Reproduction, School of Veterinary Medicine, University of California, Davis, CA, USA
d Department of Clinical Pathology, School of Medicine, Mansoura University, Mansoura, Egypt
e Center for Mass Spectrometry and Proteomics, Department of Biochemistry, Molecular Biology and Biophysics, University of Minnesota, Minneapolis, Minnesota, USA
f Department of Restorative Sciences, School of Dentistry, University of Minnesota, Minneapolis, Minnesota, USA
g Department of Diagnostic and Biological Sciences, School of Dentistry, University of Minnesota, Minneapolis, Minnesota, USA

## Slide 2
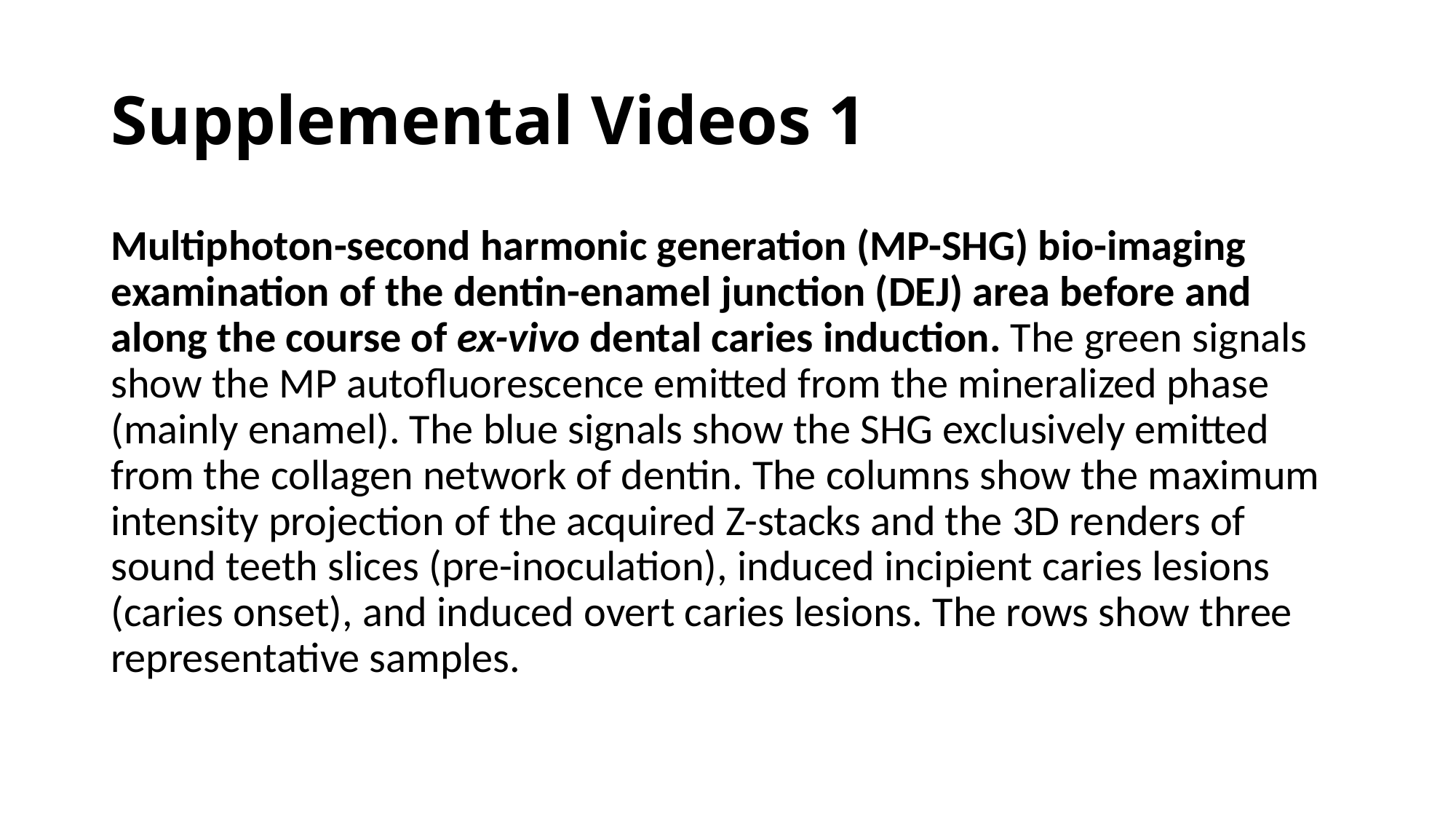

# Supplemental Videos 1
Multiphoton-second harmonic generation (MP-SHG) bio-imaging examination of the dentin-enamel junction (DEJ) area before and along the course of ex-vivo dental caries induction. The green signals show the MP autofluorescence emitted from the mineralized phase (mainly enamel). The blue signals show the SHG exclusively emitted from the collagen network of dentin. The columns show the maximum intensity projection of the acquired Z-stacks and the 3D renders of sound teeth slices (pre-inoculation), induced incipient caries lesions (caries onset), and induced overt caries lesions. The rows show three representative samples.

## Slide 3
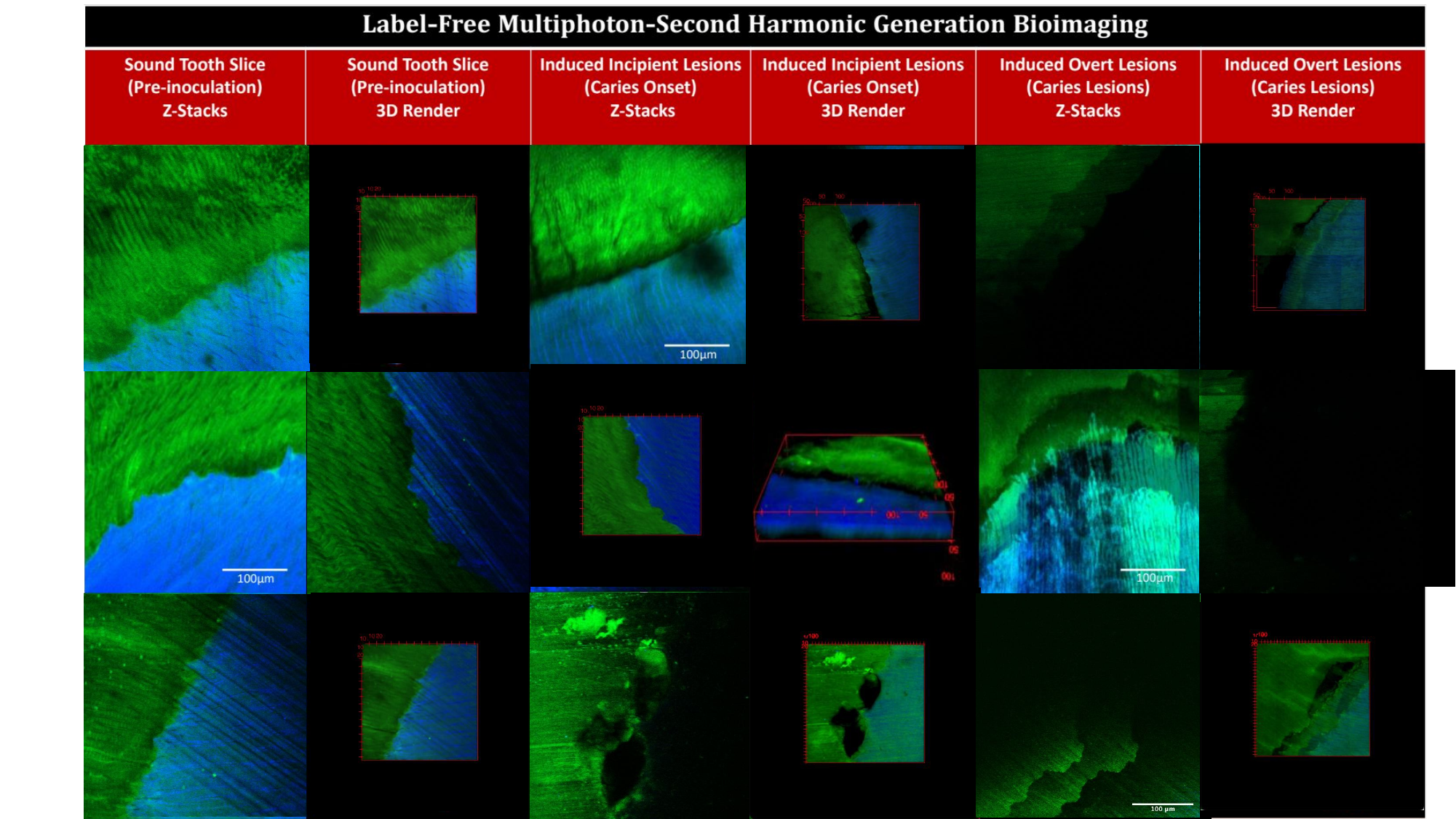

## Slide 4
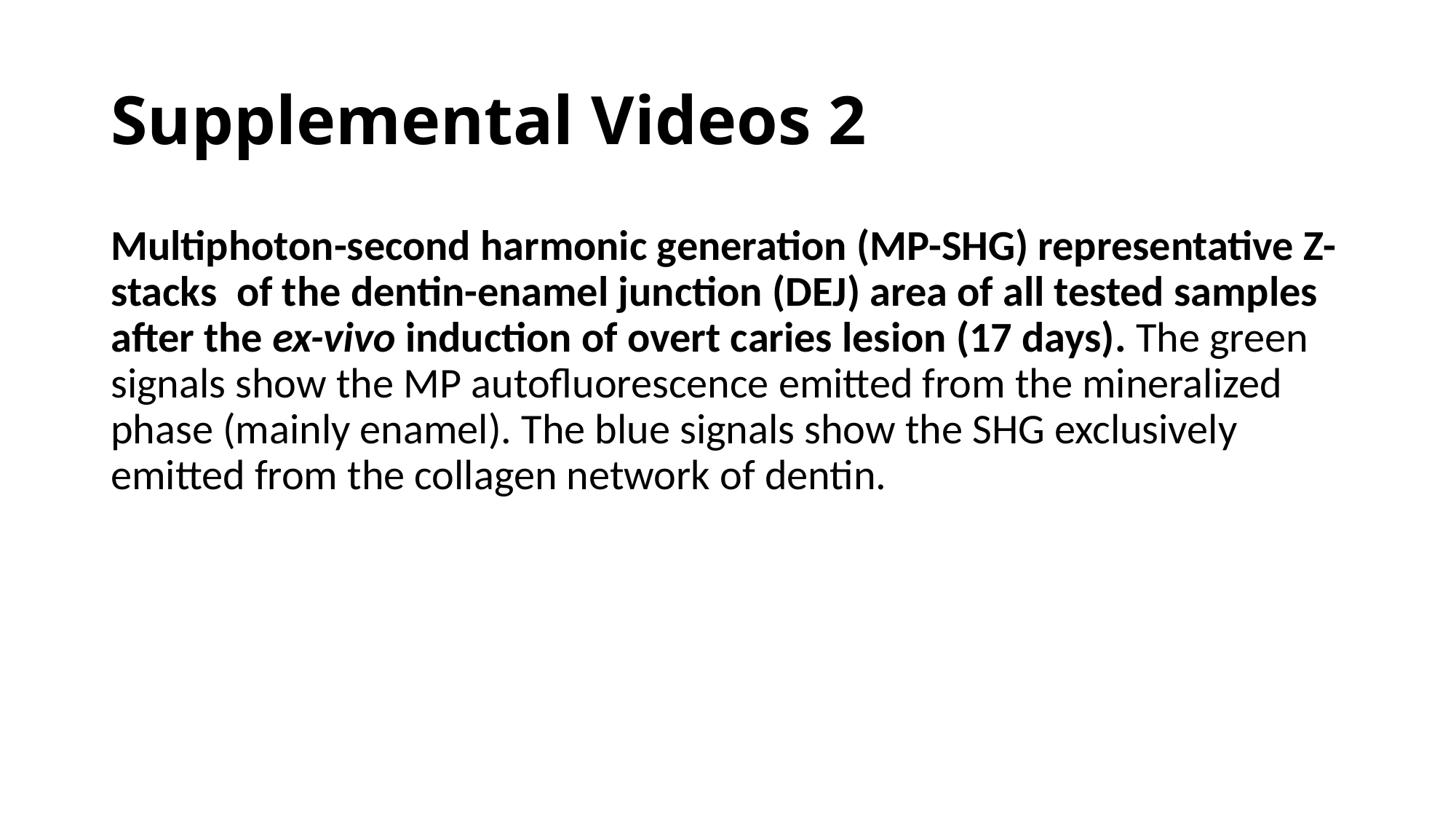

# Supplemental Videos 2
Multiphoton-second harmonic generation (MP-SHG) representative Z-stacks of the dentin-enamel junction (DEJ) area of all tested samples after the ex-vivo induction of overt caries lesion (17 days). The green signals show the MP autofluorescence emitted from the mineralized phase (mainly enamel). The blue signals show the SHG exclusively emitted from the collagen network of dentin.

## Slide 5
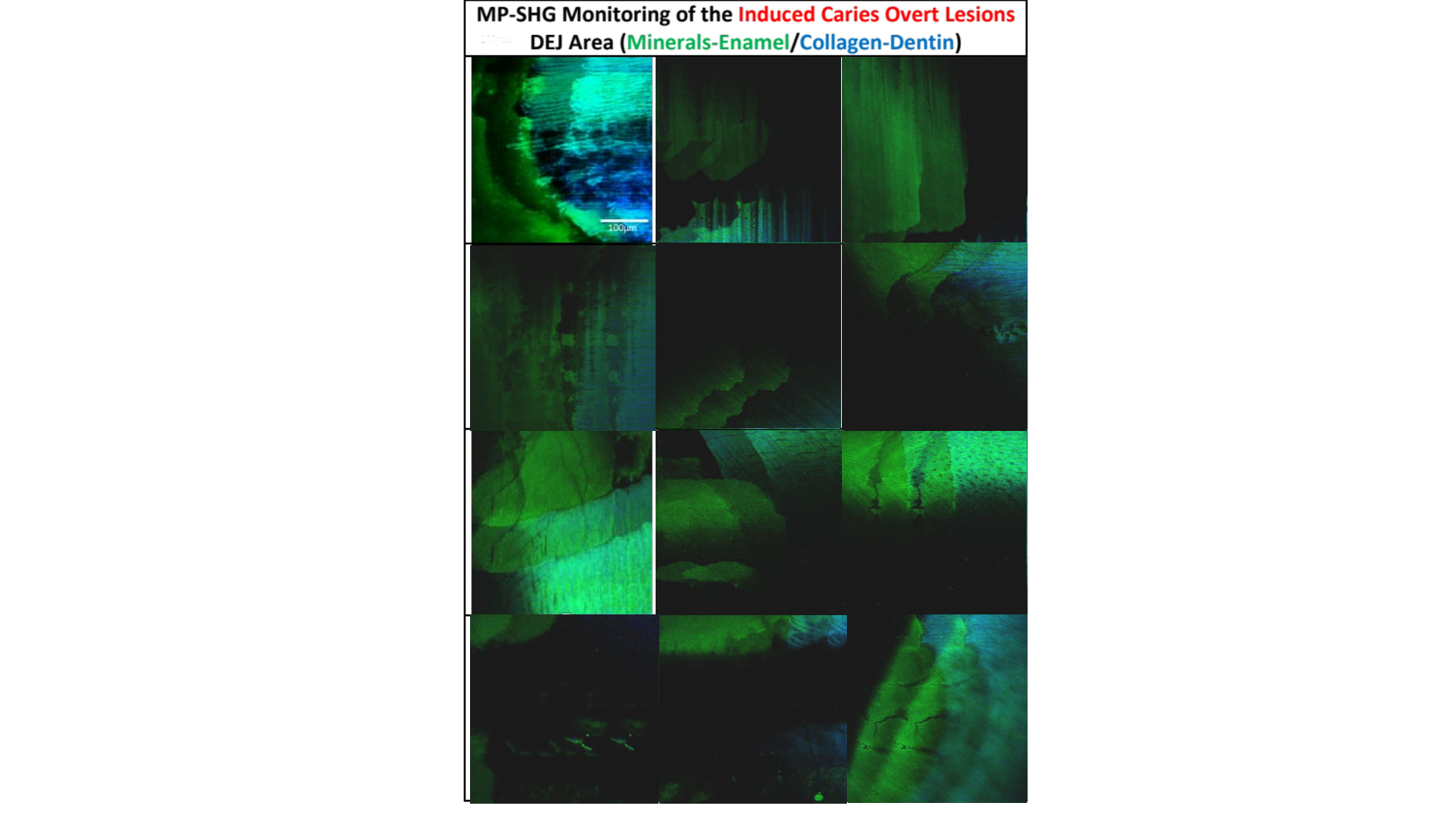

Supplement: Supplemental Material [file ZJOM_A_2123624_SM6900.zip › Supplementary files/SupplementalRichMedia.pptx]
